# Microbial enzymes as powerful natural anti-biofilm candidates

**DOI:** 10.1186/s12934-024-02610-y

**Published:** 2024-12-23

**Authors:** Lamiaa A. Al-Madboly, Asmaa Aboulmagd, Mohamed Abd El-Salam, Ivan Kushkevych, Rasha M. El-Morsi

**Affiliations:** 1 Department of Microbiology and Immunology, Faculty of Pharmacy, Tanta University, Tanta, Egypt; 2 Department of Pharmacognosy, Faculty of Pharmacy, Delta University for Science and Technology, International Coastal Road, Gamasa, 11152 Egypt; 3 School of Pharmacy and Biomolecular Sciences, Royal College of Surgeons in Ireland, Dublin, D02 VN51 Ireland; 4 Department of Experimental Biology, Faculty of Science, Masaryk University, Brno, 62500 Czech Republic; 5 Department of Microbiology and Immunology, Faculty of Pharmacy, Delta University for Science and Technology, International Coastal Road, Gamasa, 11152 Egypt

**Keywords:** Anti-biofilm, Lysostaphin, Alginate lyase, Acylase, Subtilisin, Cellobiose dehydrogenase

## Abstract

Bacterial biofilms pose significant challenges, from healthcare-associated infections to biofouling in industrial systems, resulting in significant health impacts and financial losses globally. Classic antimicrobial methods often fail to eradicate sessile microbial communities within biofilms, requiring innovative approaches. This review explores the structure, formation, and role of biofilms, highlighting the critical importance of exopolysaccharides in biofilm stability and resistance mechanisms. We emphasize the potential of microbial enzymatic approaches, particularly focusing on glycosidases, proteases, and deoxyribonucleases, which can disrupt biofilm matrices effectively. We also delve into the importance of enzymes such as cellobiose dehydrogenase, which disrupts biofilms by degrading polysaccharides. This enzyme is mainly sourced from *Aspergillus niger* and *Sclerotium rolfsii*, with optimized production strategies enhancing its efficacy. Additionally, we explore levan hydrolase, alginate lyase, α-amylase, protease, and lysostaphin as potent antibiofilm agents, discussing their microbial origins and production optimization strategies. These enzymes offer promising avenues for combating biofilm-related challenges in healthcare, environmental, and industrial settings. Ultimately, enzymatic strategies present environmentally friendly solutions with high potential for biofilm management and infection control.

## Background

There are several negative effects of bacterial biofilms on human health as well as the surrounding environment. Biofilm is the leading source of biofouling in most industrial systems and several potentially life-threatening issues in the healthcare industry. As a result, it has caused financial losses in the billions of dollars and major health concerns all over the world [1, 2].

A biofilm is a group of sessile microbial species formed on the surfaces of various natural habitats. The biofilm contains proteins, nucleic acids, and carbohydrates that nourish the growing sessile communities and keep residing cells stable [3].Water (97%) and extracellular polymeric substances (EPS), which are made up of polysaccharides, proteins, nucleic acids, lipids, mineral ions, and different cellular detritus, make up most of the biofilm matrices [4, 5]. The ability of the bacterial species to attach to surfaces and create biofilms is a crucial survival mechanism in nature [6]. Antimicrobial resistance, equipment damage, transplant operation failure, energy loss, product contamination, and the beginning of numerous chronic illnesses are all possible effects of a biofilm [7]. In addition, the majority of medical device-related biofilm infections are linked to higher morbidity, death, increased healthcare expenses, and extended hospital stays [8].

Hence, the distribution of multispecies biofilms becomes imperative. Conventional antimicrobial techniques have been demonstrated to effectively eliminate planktonic microorganisms but have failed to destroy sessile microcolonies [9]. The necessity to remove the biofilm is therefore thought to require alternative, innovative approaches [1, 10]. This review presents concise information on some important microbial enzymes that are increasingly cited to fight the formation of bacterial pathogens biofilm in healthcare, environmental, and industrial, settings. Additionally, we shed light on their microbial sources, importance, and production optimization.

### Phases of biofilm formation

**Fig. 1:**
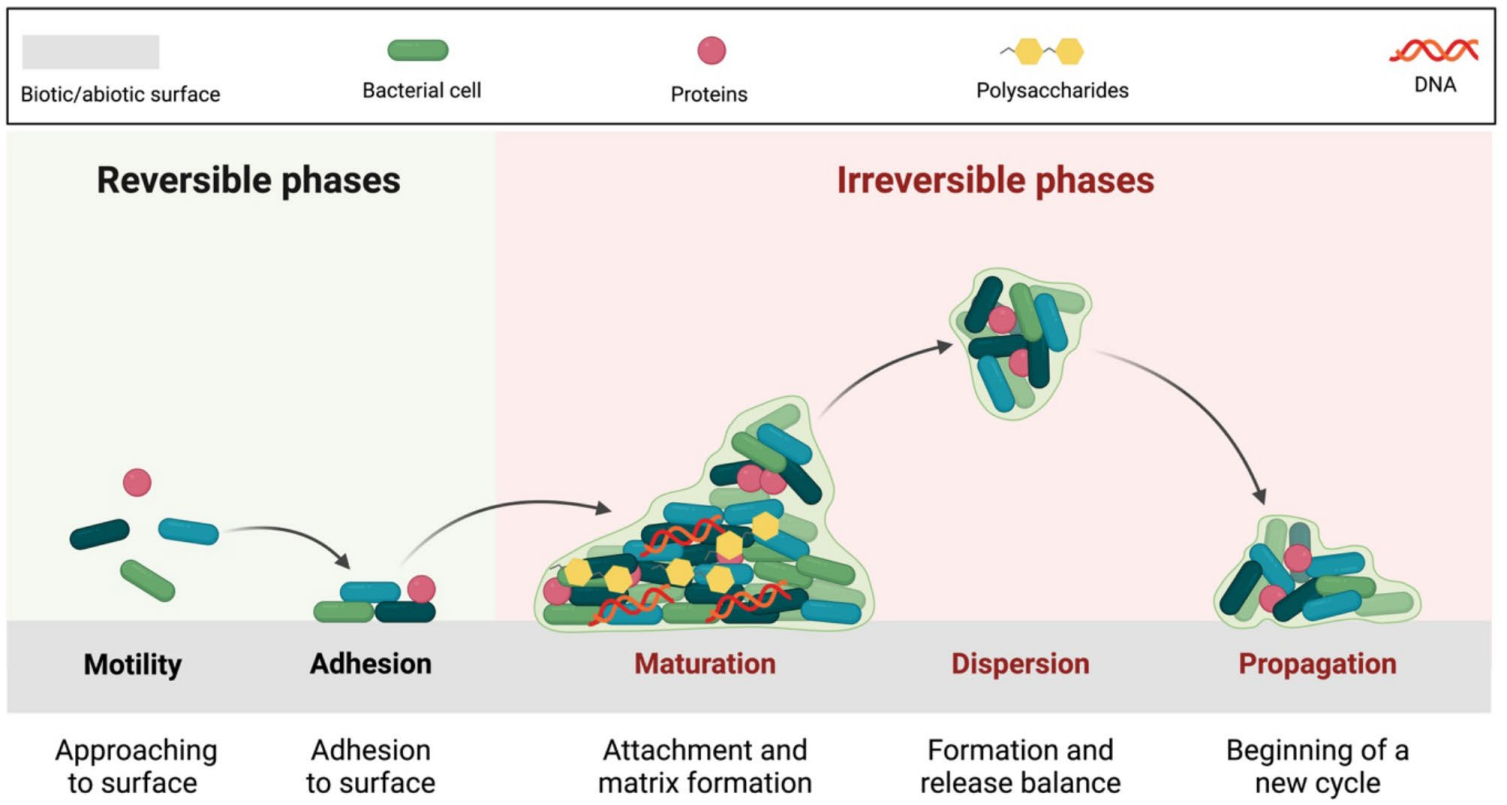
Structure and biofilm formation cycle. The biofilm formation process begins with planktonic (free-floating) bacterial cells encountering a biotic or abiotic surface. Initial stages include reversible binding, where cells weakly attach to the surface, followed by irreversible binding, where attachment becomes more stable. As the biofilm develops, microbial cells undergo growth and maturation, forming complex structures within an extracellular polymeric substances (EPS) matrix composed of DNA, proteins, and polysaccharides. This matrix protects the bacterial cells, facilitates communication, and enhances resistance to environmental stress factors

The process of creating a biofilm goes through multiple phases, the first of which is the reversible attachment and establishment of microbes to either living or non-living surfaces (Fig. 1). The hydrophobic, Van Der Waals and electrostatic bonds are responsible for the initial reversible association [11]. Common genes involved in microbial biofilm formations were summarized and presented in Table 1.

**Table 1:** Genes involved in biofilm formation phases

Phase	Gene(s) Involved	Function/Role
**Early Adhesion**	*fimbriae* (type 1), motility genes	Essential for initial cell attachment and movement [12]
**Matrix Production**	*fapABCDE*,* dksA*,* dsbA*	Involved in extracellular matrix synthesis and regulation [12, 13]
**Maturation**	*rpoN*,* brfA*,* zapE*,* truA*	Regulate gene expression for mature biofilm stability [12, 13]
**Dispersal**	*EutE*,* SufS*,* OmpL*	Facilitate biofilm dispersal and colonization [14]

The biofilm formation process starts when bacteria are exposed to certain environmental circumstances, such as iron, osmotic pressure, oxygen stress, temperature, and pH, however, the specifics of the effects of these indicators vary from one another. Exopolysaccharide synthesis and modifications to cell surface proteins are also key factors in the start of biofilm formation [15]. A bacterial microcolony is created by bacterial multiplication, which is followed by the formation of the adult biofilm, a three-dimensional structure. In the process, bacteria produce protein-based substances that essentially shield civilization from the effects of the environment. Other bacteria in the surroundings and close by can be absorbed by this matrix [16].

Cells float freely in the environment before the biofilm is formed. The bacterial surface transforms from a planktonic to a sessile state when cells start to adhere to it [17].

There are two main functions for bacterial biofilm; the bacterial cell population can be shielded against harmful foreign microbes in the first place. Additionally, it harms the host in both acute and chronic ways, preparing the body for bacterial attack and bacterial growth. Planktonic cells are more easily phagocytosed and eliminated by macrophages in the host body than are bacteria that can aggregate in biofilms, which can easily evade the host immune system and lead to chronic infections [10].

After some time, the mature biofilm breaks up, and liberated bacterial cells might create new biofilm foci in other places. Different bacterial species have unique characteristics that affect how they break away from biofilms and disseminate to neighboring habitats. Cells disperse within the biofilm in uropathogenic *Escherichia coli* bacteria in response to an increase in extracellular iron content. Increasing the concentration of carbon and nitrogen would affect the biofilm dispersion in *Pseudomonas aeruginosa* [18, 19].

Exopolysaccharides serve as binding platforms for proteins, lipids, nucleic acids, and other molecules to attach to various surfaces. Finding a good solution to this dilemma is important since the prevalence of resistance to standard antibiotic therapy is rising [20].

Beyond the formation of bacterial biofilms, there is a mechanism called the quorum sensing system that aids in cell communication and activates genes involved in the creation of virulence factors [1].

Because the quorum signaling system controls the production of virulence factors and the formation of biofilm, its disruption would prevent microbial infections, which has given rise to a strong motivation to develop medicines based on the suppression of bacterial quorum sensing [21].

### Targeting biofilm

Using antibiotics to eradicate the bacteria in biofilm faces several challenges. A few of the issues that impede antibiotics from killing bacteria within the biofilm include; restricting antibiotic penetration into the biofilm and medication loss due to the activity of damaging enzymes. Antimicrobials can be resisted by biofilms up to 1,000 times more than they can by planktonic organisms. For biofilm eradication, factors that prevent biofilm development or cause bacteria to do transition from biofilm-mode to free life are optimal [3, 10].

**Fig. 2:**
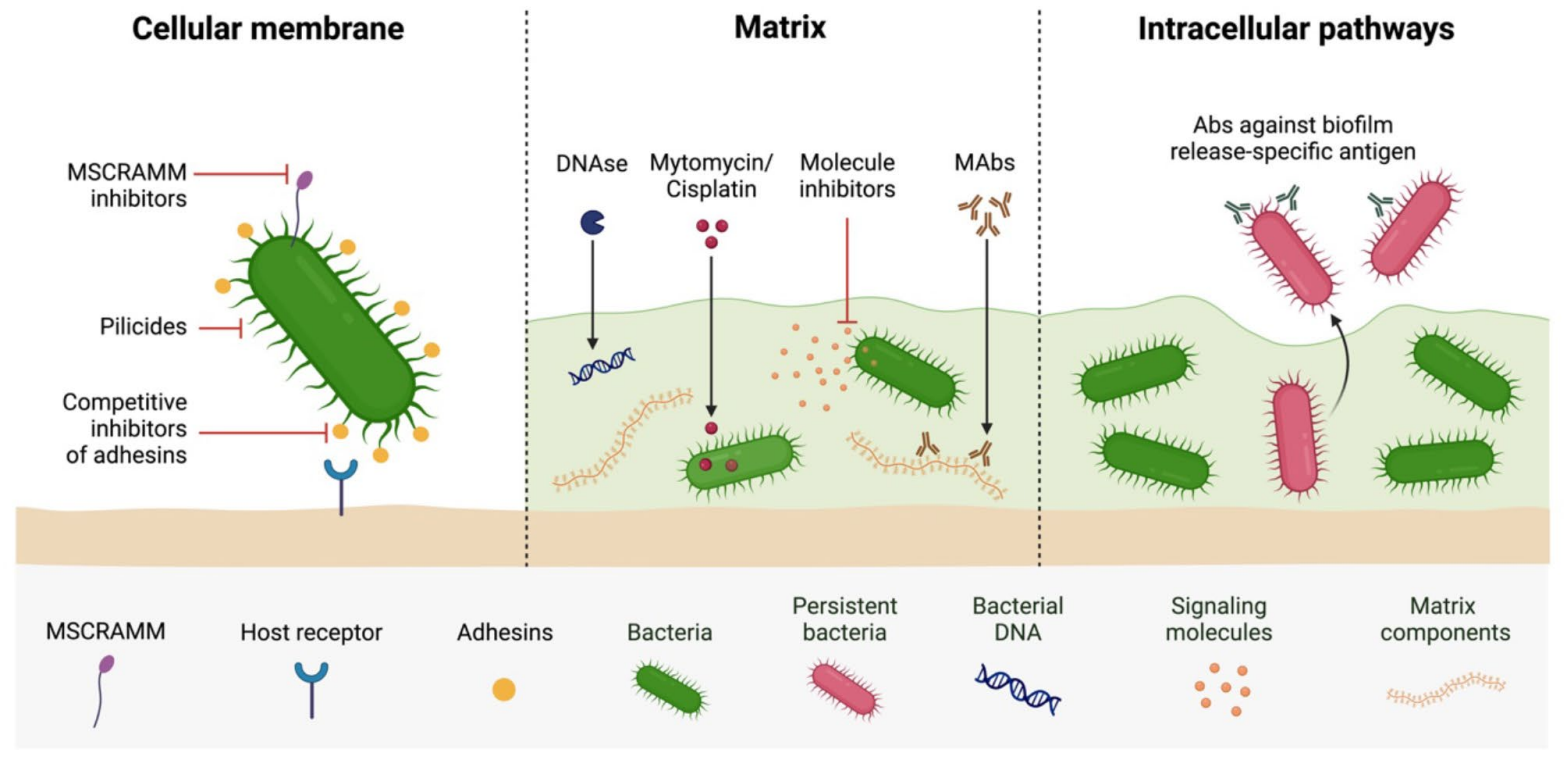
Anti-biofilm therapeutic target sites organized by cellular membrane, matrix, and intracellular pathways. Anti-biofilm strategies target distinct components of biofilm structure and bacterial cells to disrupt biofilm formation and enhance bacterial susceptibility. At the cellular membrane, inhibitors of adhesive matrix molecules (MSCRAMM) and competitive adhesion block bacterial surface structures; pili or fimbriae, to prevent attachment and biofilm formation. Quorum sensing inhibitors (QSIs) and acylase enzymes interfere with membrane-based communication by disrupting signaling molecules like acyl-homoserine lactones (AHLs), which are essential for biofilm coordination. Iron chelators also act at the membrane level, restricting access to iron, which is crucial for biofilm stability and growth. In the biofilm matrix, matrix-degrading enzymes target the extracellular scaffold, including polysaccharide-degrading enzymes like dispersin B, α-amylase, and alginate lyase, which weaken the structural integrity of the biofilm. DNase I degrades extracellular DNA (eDNA), a component vital for matrix cohesion, while the proteases: proteinase K and trypsin digest extracellular proteins, destabilizing the matrix and making embedded bacteria more accessible to treatments. Dispersal inducers, like cis-2-decenoic acid and D-amino acids, promote biofilm dispersal by releasing bacteria into a planktonic state, where they are more susceptible to treatment. Within intracellular pathways, metabolic inhibitors target the biofilm-specific metabolic activities; fatty acid and LPS synthesis, reducing biofilm resilience. Immune-based strategies, including phagocytosis enhancers and biofilm-specific antibodies, help immune cells recognize and eliminate biofilm-associated bacteria. Lysozyme disrupts bacterial cell walls, while cellobiose dehydrogenase indirectly affects biofilm stability by targeting polysaccharide-rich biofilms. These combined strategies weaken biofilm defenses and promote clearance

The three primary strategies used by anti-biofilm agents include targeting cellular membrane, matrix, and intracellular signaling pathways (Fig. 2). First, regulation of matrix synthesis and its regulatory mechanisms (QS system). As an example, the pre-binding of bacteria to surfaces, which is dependent on the assessment of adhesins, appears to be significant and effective in reducing the stability of biofilms. while digestion of the EPS matrix may be another method of interfering with the formation of biofilms [10].

### Microbial enzymes as antibiofilm agents

Biofilm forming bacteria are encased in the extracellular matrix, hence one way to compromise the integrity of the biofilm is by matrix degradation. This can be done by using degrading enzymes such as glycosidases, proteases, and deoxyribonucleases [10]. Enzymes can regulate the biofouling process [22] by eliminating different kinds of biomolecular coatings and proteins from different biotic and abiotic surfaces [23].

The use of a complex enzyme formulation that contains DNase to degrade extracellular DNA, CDH to hydrolyze extracellular polysaccharides, proteases to hydrolyze proteins, and anti-quorum sensing enzymes to prevent biofilm formation is necessary for the successful removal of complex biofilms [24]. The control of biofilms using such enzymes can be considered as an environmentally eco-friendly strategy due to their non-toxic characteristics and biodegradability [25, 26]. Additionally, numerous enzymes produced by bacteria, fungi, and algae have the power to alter the structure of biofilms by destroying the exopolysaccharide, extracellular DNA, and protein content of EPS or by interfering with quorum sensing [27]. Figure 2 and Table 2 present some enzymes which reported to exhibit antibiofilm activities [10].

**Fig. 3:**
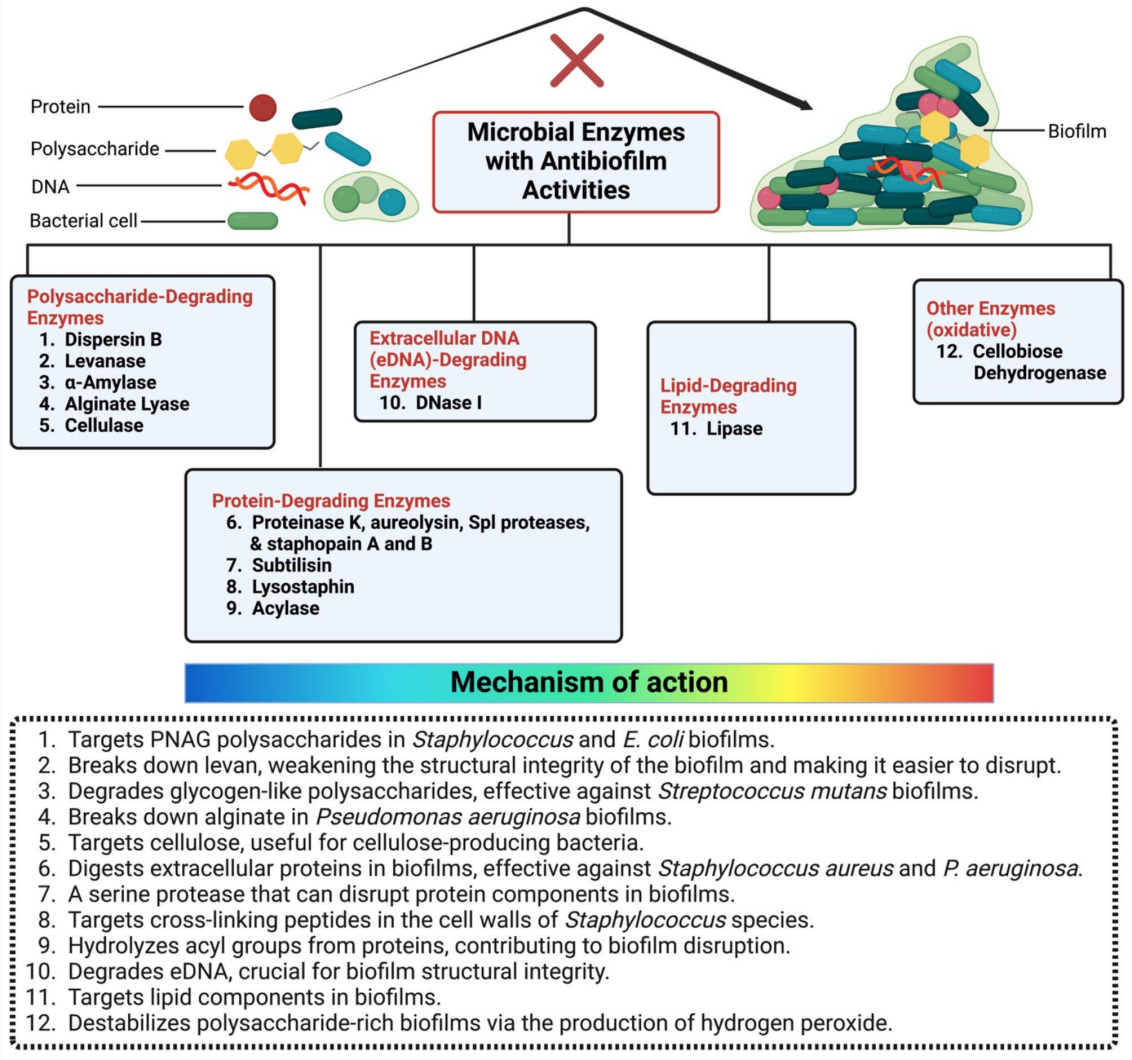
Classification of main microbial enzymes with anti-biofilm activities and their modes of action. This figure categorizes various enzymes known for their ability to disrupt biofilms and outlines their specific mechanisms of action. Enzymes are classified based on their targets within the biofilm matrix, including polysaccharides, proteins, and extracellular DNA (eDNA). The mode of action of each enzyme is illustrated, highlighting how they degrade biofilm components, weaken structural integrity, and enhance the susceptibility of embedded bacteria to antimicrobial agents

**Table 2:** Some of the most important enzymes that are stated for their antibiofilm activities against certain microbial pathogens

Class	Enzyme	Target biofilm	Mechanism of action	Reference
Extracellular DNA (eDNA)-degrading enzymes	eDNase I	*P. aeruginosa* and *Staphylococcus aureus*	Degrades eDNA leading to the inhibition of biofilm formation	[28]
Protein-degrading enzymes (Proteases)	Proteinase K	Effective against *S. aureus* and *P. aeruginosa*	Digests extracellular proteins in biofilms	[29]
	Subtilisin	*Escherichia coli*	A serine protease that can disrupt protein components in biofilms	[30]
	Lysostaphin	*S. aureus*	Degrading the bacterial cell wall	[31]
	Acylase	*P. aeruginosa*	The inhibition of biofilm formation	[32]
	Protease	*Candida albicans*	Degrading biofilm	[33]
Polysaccharide-degrading enzymes	Levanase	*S. aureus* and *P. aeruginosa* biofilms	Degrades levan, weakening the structural integrity of biofilm and making it easier to disrupt	[34]
	Dispersin B	*Staphylococcus epidermidis* and *E. coli*	Targets poly-N-acetylglucosamine (PNAG) polysaccharides	[35]
	α-Amylase	Different strains of multi-drug resistant bacteria such as *Streptococcus mutans* biofilms	Degrades glycogen-like polysaccharides	[36]
	Alginate lyase	*P. aeruginosa* Biofilm-grown *Helicobacter pylori*	Breaks down alginate in the biofilm structure The degradation of polysaccharides in EPS	[37]
	Cellulase	Cellulose-producing bacteria such as *P. aeruginosa*,* Salmonella enterica*, *nterococcus faecalis*	Targets cellulose	[38]
Lipids-degrading enzymes	Lipase	*E. coli* and *S. aureus*	Targets lipid components in biofilms	[39]
Other enzymes (oxidative)	Cellobiose dehydrogenase	*S. aureus*	Destabilizing polysaccharide-rich biofilms by generating hydrogen peroxides, leading to degradation of biofilms	[40]

### Polysaccharide-degrading enzymes

#### Dispersin B

The extracellular matrix of most bacterial biofilms is composed of polysaccharides, proteins, and nucleic acids. These biopolymers play crucial roles in key biofilm-related characteristics, such as attachment to surfaces, intercellular adhesion, and resistance to biocides. Enzymes that break down the polymeric components of the biofilm matrix including glycoside hydrolases, proteases, and nucleases are valuable tools for investigating the structure and function of these components. They are also being explored as potential anti-biofilm agents for clinical applications. One notable example is Dispersin B, a well-characterized, broad-spectrum glycoside hydrolase produced by *Aggregatibacter actinomycetemcomitans* [35]. Dispersin B targets poly-N-acetylglucosamine (PNAG), a polysaccharide in the biofilm matrix that facilitates biofilm formation, stress resistance, and biocide tolerance in various Gram-negative and Gram-positive pathogens. This enzyme has demonstrated the ability to inhibit the formation of biofilms and pellicles, detach established biofilms, disaggregate bacterial clusters, and increase the susceptibility of preformed biofilms to detachment by enzymes, detergents, and metal chelators, as well as to destruction by antiseptics, antibiotics, bacteriophages, macrophages, and predatory bacteria. Findings from nearly 100 in vitro and in vivo studies conducted on Dispersin B since its discovery two decades ago were reported [35].

#### Cellulase

A study was conducted to explore the effectiveness of cellulase in inhibiting biofilm formation by *P. aeruginosa*, a pathogen frequently found in medical implants. In this experiment, a biofilm was cultivated on glass slides within a parallel flow chamber for four days, using glucose as the nutrient source. Biofilm development was evaluated by measuring colony-forming units (CFU) and biomass areal density. The biofilms were grown at pH levels of 5 and 7, with three different cellulase concentrations: 9.4, 37.6, and 75.2 units/mL. A control experiment with inactivated cellulase was also included. The findings demonstrated that cellulase effectively reduced biomass and CFU formation by *P. aeruginosa* on glass surfaces. The extent of inhibition was dependent on the concentration of cellulase and was more pronounced at pH 5 compared to pH 7. Furthermore, the investigation extended to assess how cellulase affected the apparent molecular weight of purified *P. aeruginosa* exopolysaccharides (EPS). Results from size exclusion chromatography indicated a reduction in the apparent molecular weight of EPS when incubated with cellulase. Additionally, there was an increase in the amount of reducing sugars over time when the purified EPS were treated with cellulase, supporting the notion that cellulase degrades *P. aeruginosa* EPS. Although cellulase did not completely prevent biofilm formation, it suggests potential for use in combination with other treatments or enzymes to enhance anti-biofilm efficacy [38].

Previous studies have highlighted the challenges posed by biofilm-associated infections, particularly those caused by *P. aeruginosa*. The combination of ceftazidime and cellulase has shown significant anti-biofilm effects, including the inhibition of biofilm formation and the eradication of established biofilms in this pathogen. These data suggest that glycoside hydrolase therapy represents a novel strategy with the potential to enhance the efficacy of antibiotics and help resolve biofilm-associated wound infections caused by *P. aeruginosa* [38].

### Levan hydrolase

Levan is an extracellular polysaccharide with a high molar mass that is produced by a variety of bacteria either as a slime extruded into the growth media or as a capsule connected to the cell wall. When this exopolysaccharide (slime), a moist and sticky substance, occurs during the production of paper, it helps the cells to connect to one another and to entrap debris, finally resulting in the formation of slime deposits. These lead to manufacturing losses and ultimately drive up the price of final products [41].

Freis (1984) employed a method for the levanase enzyme, a non-toxic enzyme that hydrolyzes slime, to dissolve slime deposits of Levan. By removing slime from the system, the enzyme treatment exposes microbial cells directly to biocides at lower levels and, as a result, indirectly contributes to fewer environmental issues [42].

Levansucrase, a member of the glycoside hydrolase family 68 (GH68), has been discovered in a range of microbes, including certain lactic acid bacteria (LAB), Bacillus and Pseudomonas species. Diverse thermophilic bacteria also provide a source of levan hydrolase [42]. Also, *Rhodotorula* sp. is a significant member of a large group of bacterial and filamentous species that are known for their capacity to break down Levan [41].

*Rhodotorula* sp. produced a high yield of levanase (12.5 nkat/mL) in shake flasks in basal medium containing 1% maltose as the sole carbon source. Maltose was found to be the best carbon source for levanase production among the several sources studied. Levanase optimum production requires a temperature of 30 °C and a pH of 6. The enzyme production was higher in a batch reactor than it was in shaken flasks [41].

Analytical methods: Levan-hydrolase activity was assayed according to Takahashi (1983). Levan was dissolved in 20 mmol/L phosphate buffer, pH 6.5, and was added to a 0.5 mL suitably diluted enzyme preparation. The mixture was then incubated at 40 °C for 30 min [41]. The next step is a qualitative TLC analysis of the sugars in the reaction mixture to confirm the existence of levanase activity. The reducing sugars in the reaction mixtures can then be measured using either the arsenomolybdate method (Somogyi 1952) or the Dinitro Salicylic Acid (DNS) Method for a quantitative assessment of enzyme activity [41, 42]. The quantity of enzyme that released 1 nmol of reducing sugar, which is equivalent to fructose per second, was used to define one unit of levanase activity [41].

### Alginate lyase

A frequent antibiofilm substance produced by bacteria associated with alginate-rich algae is alginate lyase. Alginate, the primary element of biofilm, can be effectively depolymerized by the enzyme. Searching for bacteria that produce the alginate lyase enzyme in the marine environment has led to the identification of useful species of bacteria that produce alginate lyase and, as a result, new varieties of alginate lyase enzymes [43].

According to reports, alginate, sometimes referred to as algal polysaccharide, is the main extracellular polymeric material (EPS) in biofilms. As a result, targeting alginate as a key component of biofilms has emerged as a promising therapeutic approach for treating bacterial infections involving biofilms and associated antibiotic resistance. Numerous studies have looked at using bacterial alginate lyase as an antibiofilm agent to combat biofilms formed by harmful bacteria as *P. aeruginosa*, *Helicobacter* sp. and *Enterococcus* sp., *Streptococcus pneumoniae*, *Haemophilus influenza*, *S. aureus*, *Klebsiella pneumoniae*, *Acinetobacter baumannii*, *Enterococcus faecalis*, and *E. coli* [43].

Recent research has shown that a purified alginate lyase (AlyP1400) from a marine *Pseudoalteromonas* sp. 1400 bacterium is capable of disrupting the formation of *P. aeruginosa* biofilms by decomposing alginate within the extracellular polysaccharide matrix, hence increasing tobramycin’s bactericidal action, which may be a feasible approach for combination treatment [44].

Many types of organisms, including algae, marine mollusks, marine and terrestrial bacteria, certain viruses, and fungi have alginate lyase that has been isolated from them [43]. Marine bacteria, the main producers, were shown to have the greatest diversity of alginate lyases [44]. A marine bacterium called *Pseudoalteromonas*, which generates exoproducts like alginate lyase, has shown antibacterial effectiveness against a variety of pathogens, including *Salmonella enterica*, *S. aureus*, *P. aeruginosa*, *Escherichia coli* and *E. faecalis* and antibiofilm activity against *S. enterica*, *P. aeruginosa* and *E. coli* [45].

Production of Alginate Lyase by *Pseudoalteromonas* sp. 1400: For the microorganism isolation, a selection medium containing 5.0 g/L sodium alginate, 5.0 g/L (NH4)2SO4, 2.0 g/L K2HPO4, 30.0 g/L NaCl, 1.0 g/L MgSO4·7H2O, and 0.01 g/L FeSO4·7H2O at pH 7.0 and employing sodium alginate as the only carbon source was utilized. At pH 8.0, *Pseudoalteromonas* sp. 1400 produced the highest amount of alginate lyase, with a specific activity of 32.85 U/mg protein and an overall activity of 56.5 0.71 U/ml [46]. Based on the hydrolytic clearing zone diameter created after adding Lugol solution to alginate plates, alginate lyase activity was identified.

### α-Amylase

One of the most significant industrial enzymes, amylase holds the largest market share for enzyme sales and finds extensive use in the pulp and paper industry, analytical chemistry, detergents, textile desizing, starch industry, baking, and detergents sectors. These amylase enzymes come from a variety of sources, including plants, animals, and microbes, Because of their high productivity and thermostability, microbial amylases are the most manufactured and employed in industry [47]. Amylases can hydrolyze the polysaccharide structure of EPS, which makes them effective for managing biofilms. Amylase, particularly α-, β-, and amyloglucosidase, would be included in the combination of enzymes to lessen the buildup of biofilm on diverse biotic and abiotic surfaces [3].

Alpha-amylase enzyme has recently been discovered to be an effective antibiofilm agent against the clinical pathogens *Vibrio cholera*, *S. aureus*, and *P. aeruginosa*. In microtiter plate assays and congo red assays, the amylase enzyme demonstrated remarkable antibiofilm action against the marine-derived bacteria *P. aeruginosa* and *S. aureus* [47].

*Bacillus amyloliquefaciens*, *Bacillus licheniformis*, *Bacillus stearothermophilus*, *Bacillus subtilis*, and *Bacillus cereus* are often the sources of the enzyme α-amylase in bacteria. The amylases produced by various Bacillus species differ not only in type (saccharifying or liquefying), but also in the pH and temperature ranges that they can withstand [47].

At temperatures between 37 and 60 °C, the bacteria *B. amyloliquefaciens*, *B. subtilis*, *B. licheniformis*, and *B. stearothermophilus* are employed to generate their α-amylase. *B. cereus* (strain obtained from deep sea water sample) generated a thermostable amylase enzyme that had an optimal temperature for production of 50 °C. *Bacillus* sp., which was isolated from soil, found that the amylase enzyme could be generated most efficiently at 90 °C showing 100% activity. *Bacillus cereus* strain produced the most amylase enzyme when the pH was 11, while the optimum pH was 10.5 [47, 48].

### Protein-degrading enzymes (proteases)

Proteases are protein hydrolyzing enzymes. There are many different types of enzymes in this category, each with a unique structure, target substrate, reaction mechanism, and set of physicochemical characteristics. Exopeptidases and endopeptidases are the two main families of proteases. They may also be divided into three groups based on the pH of their optimal conditions: acidic, neutral, or alkaline [49].

Microbial proteases, especially those from the *Bacillus* genus, are the most widely used commercial enzymes and are employed extensively in the production of detergent and other industrial products. Proteases have the potential to be used as antimicrobial agents for numerous illnesses and infections in addition to being proteolytic enzymes, which might have a significant influence on various clinical treatments [50].

Protease enzymes are produced by a variety of microorganisms and are thought to have a role in both the control of metabolism and the emergence of several infectious diseases. Since proteins make up a large portion of biofilms, proteases are thought to be the best enzymes for eliminating them. Proteinase K, aureolysin, Spl proteases, and staphopain A and B are only a few of the proteases that different microbes produce to break down biofilms [50]. Among the most significant proteases utilized as antimicrobial and antibiofilm agents are *Bacillus sp. subtilisin*, *Staphylococcus* lysostaphin, and Bacteriophage lysins [49].

In order to create anti-biofilm surfaces based on non-toxic chemicals and sustainable methods, proteases were immobilized on a polypropylene surface inhibited the adherence of *C. albicans* biofilms [10].

The *Bacillus* genus produces high yields of neutral and alkaline proteolytic enzymes with remarkable properties, including high stability toward extreme temperatures, pH, organic solvents, detergents, and oxidizing compounds. *Bacillus* is likely the most significant bacterial source of proteases [51].

Most extracellular proteases produced by Bacillus are serine proteases (i.e., *B. subtilis*, *B. pumilus*), cysteine proteases (i.e. *B. licheniformis*) and metalloproteases (i.e. *B. stearothermophilus*) [51]. New proteolytic *Bacillus alkalitelluris* TWI3(Alkaline Metalloprotease) was discovered and its ability to produce proteases was examined. Maximum production of protease was achieved using lactose as a carbon source, skim milk as a nitrogen source and optimum growth temperature was found to be 40 °C at pH 8 [52].

### Proteinase K

Proteinase K, a broad-spectrum serine protease, is capable of digesting extracellular proteins in biofilms and is effective against *S. aureus*,* L. monocytogenes* and *E. coli* [29, 53–55].

A study by Sugimoto et al. [29] demonstrated that treating clinical isolates of *S. aureus* with proteinase K significantly inhibited biofilm formation in 13 out of 17 isolates, with reductions exceeding 90%. Additionally, the treatment degraded the extracellular polymeric substances (EPS), leading to a marked reduction in preformed biofilms in 14 out of 17 isolates, with decreases of up to 80%. In a separate investigation, Nguyen and Burrows [53] found that a 5-minute exposure to proteinase K resulted in less than 20% residual biofilm across all tested biofilms of three *L. monocytogenes* strains. Similarly, reductions in biofilm thickness of *S. aureus* and *E. coli* O157 were reported by Shukla and Rao [54] and Lim et al. [55], respectively, using confocal laser scanning microscopy (CLSM) after proteinase K treatment.

Eladawy et al. [56] conducted experiments to assess the impact of some enzymes on biofilm disruption and inhibition. Proteinase K, was evaluated for its potential to degrade protein components of the biofilm matrix, whereas Lysozyme, a naturally occurring enzyme with bacteriolytic properties, was tested for its ability to degrade the peptidoglycan layer. Cephalosporins, a class of β-lactam antibiotics, were included to determine their effectiveness against biofilm-associated bacteria when combined with these enzymes. They demonstrated that lysozyme and Proteinase K significantly reduced biofilm formation when used alone and exhibited enhanced effects when used in combination. The study suggests that combining enzymatic treatment with antibiotics could be a promising strategy to combat biofilm-associated infections caused by *P. aeruginosa*, offering potential improvements in the management of chronic and persistent infections.

Yang and colleagues [57] aimed to enhance the yield of Proteinase K by employing multi-copy expression strains of *Pichia pastoris*. They constructed a recombinant strain containing multiple copies of the *Proteinase K* gene integrated into the yeast’s genome. The expression vector used was designed to include the AOX1 promoter, known for its strong induction in the presence of methanol. By optimizing the expression conditions, including varying methanol concentrations and controlling pH levels, the researchers achieved a substantial increase in enzyme production. The study demonstrated that multi-copy integration significantly improved the expression level of Proteinase K compared to single-copy strains. The resulting recombinant Proteinase K exhibited high enzymatic activity, comparable to its commercially available counterparts. Furthermore, the purification process was streamlined, yielding a high-quality enzyme suitable for various biotechnological applications.

### Subtilisin

Subtilisin, a serine protease, is known for its relatively broad and non-specific enzymatic activity. Several studies have shown that subtilisin is an effective enzyme for the disruption of biofilms [58]. For instance, one study found that treatment with subtilisin led to the most significant reduction in colony-forming units (CFUs) of *Pseudomonas fluorescens* biofilm when compared to enzymes targeting polysaccharides or lipase [59]. In another investigation, subtilisin demonstrated greater efficacy than polysaccharide-degrading enzymes such as amylase in eliminating the biofilm of *Macrococcus caseolyticus* isolated from dairy environments [60]. Additionally, subtilisin was identified as the most effective enzyme in reducing the inter-kingdom multispecies biofilm model composed of *S. aureus*, *E. coli*, and *C. albicans* [58].

The study of Shreya et al. [61] investigated the production and optimization of subtilisin, a protease enzyme, from *B. subtilis* strain ZK3, alongside the biological and molecular characterization of subtilisin-capped nanoparticles. The primary objective was to enhance the yield of subtilisin by optimizing various cultivation parameters such as temperature, pH, incubation time, and substrate concentration. The researchers employed statistical methods, including response surface methodology (RSM), to determine the ideal conditions for maximum enzyme production. The results indicated that optimal subtilisin production was achieved at a temperature of 37 °C, pH 7.0, and an incubation time of 48 h, with specific carbon and nitrogen sources significantly influencing enzyme synthesis.

### Lysostaphin

Lysostaphin is an antibacterial enzyme which is specifically capable of cleaving the cross-linking pentaglycine bridges in the cell walls of staphylococci [62]. *S. aureus* cell walls contain high proportions of pentaglycine, making lysostaphin a highly effective agent against both actively growing and quiescent bacteria. Lysostaphin, the pentaglycine endopeptidase, is a zinc metalloproteinase [63] which is widely used to eradicate susceptible Staphylococcus aureus and *Staphylococcus epidermidis* biofilms [64] that are significant issues in healthcare settings, particularly for patients who are immunosuppressed and immunocompromised and who have indwelling devices [65].

Lysostaphin has been widely used in research laboratories as a substance to distinguish between different species of Staphylococci and to lyse staphylococcal cell walls in order to release internal enzymes, nucleic acids, cell membranes, and surface components [66].

According to several studies, lysostaphin is a potential agent for eliminating staphylococci biofilm from biotic and abiotic surfaces, including medical devices like catheters. The most recent therapeutic usage for lysostaphin is its immobilization on the surface of meshes or wound dressing materials used in herniorrhaphy [63].

Interest in lysostaphin as a treatment for staphylococcal infections has increased [67, 68] due to the escalating spread of *S. aureus* that is resistant to antibiotics. Lysostaphin activity makes antibiotic-resistant *S. aureus* organisms, such as methicillin resistant *S. aureus* (MRSA) and intermediately vancomycin susceptible S. aureus, susceptible [69].

In 1964, Schindler and Schuhardt became the first to isolate lysostaphin from *Staphylococcus simulans biovar staphylolyticus* [62]. The enzyme is still not commonly employed in medicine, veterinary medicine, or as a food preservative despite having several benefits and showing promising preliminary study results. The high manufacturing costs of the enzyme are one of the most significant constraints preventing its use in clinical or technical settings [63].

Therefore, increased lysostaphin production was extensively researched. Lysostaphin has so far been cloned and expressed in several expression host cells and systems [70].

Cells of the *E. coli* TOP10F’ strain (Invitrogen, USA) transformed with the plasmid pBAD2Lys were used to produce the recombinant lysostaphin. The initial optimization of production of recombinant lysostaphin was performed using LB medium. A single colony of pBAD2Lys-transformed *E. coli* TOP10F’ was introduced into 300 mL of ampicillin-containing media. At 37 °C, the bacteria were grown overnight in an aerobic condition on a rotator shaker (200 rpm). The resulting cell suspension was added to a bioreactor that held 2.7 L of sterile LB medium supplemented with 100 mg/L of ampicillin [50]. For optimization of fermentation conditions, previous studies showed that the following conditions: pH 6.0, temperature 37 °C, and mechanical stirrer speed 400 rpm could produce the highest amount of recombinant lysostaphin in a bioreactor [61, 62].

A change in the growing medium’s composition led to an even greater increase in the enzyme production efficiency: Three different media were tested: enriched LB medium, high cell density cultivation (HCDC) medium, and LB medium (containing three distinct glycerol feeding techniques of enriched LB medium), Only in the case of enriched LB medium supplemented with glycerol at the rate of 3 g/Lxh, initiated at the time of induction, was a favorable impact seen as follow: The specific activity yield of the enzyme has increased by almost twice after extra glycerol administration (25923 U/L), which was mostly the result of more biomass being generated (66% more than on LB medium) [63].

According to Marova and Kovar, lysostaphin’s bacteriolytic activity was assessed using a spectrophotometric assay [71] with few modifications: The reaction mixture was preincubated at 37^o^C for 10 min with a suspension of *S. aureus* ATCC 29,213 cells in 0.1 M phosphate buffer (pH 7.5) to an OD600 of 0.25 and after that, various amounts of lysostaphin solutions were added. The changes in turbidity of the reaction mixture were assessed following 10 min of incubation at 37 °C. A preparation was considered to have one unit (U) of lysostaphin activity if it reduced the turbidity of a 6 mL cell suspension by 50% in just 10 min at 37ºC [63].

### Quorum-quenching enzymes

Anti-quorum sensing enzymes are one of the newly discovered classes of antimicrobial enzymes. As clarified before in this review, bacteria use quorum sensing to control a variety of physiological processes including; bacteriocin and antibiotic production, conjugation, motility, competency, virulence and spore, and development of biofilms [72, 73]. Among the compounds suggested as targets are acyl homoserines lactones (AHLs), which have an important role in controlling the pathogenicity of bacteria in more than fifty different species, Furthermore, the most well-studied quorum-sensing molecules are AHLs. These chemicals that sense quorums flow in and out of the cell by either active or passive diffusion conveyance. But scientists have also found enzymes that quench quorum, like lactonases which cause hydrolysis of the ester bond of the “homoserine lactone ring” for the acylated homoserine lactones. Therefore, they could prevent AHLs from their binding to specific target transcriptional regulators. Additionally, the first anti-quorum-sensing enzyme encoded by *aiiA* gene was extracted from a soil bacterial strain that belongs to *Bacillus* sp [73].

### Acylase

Quorum-quenching acylases are enzymes that degrade *N*-acyl-L-homoserine lactones (AHLs), signaling molecules involved in bacterial communication. By interfering with this signaling, these enzymes can inhibit undesirable bacterial behaviors such as biofilm formation and virulence factor production, making them promising candidates for controlling bacterial infections [74].

Sompiyachoke and co-workers [74] tried to engineer quorum-quenching acylases, specifically focusing on two AHL acylases enzymes; PvdQ and MacQ, to enhance their biochemical properties and kinetic efficiency. They employed a novel time-course kinetic assay to improve the activity of these acylases. For PvdQ, engineering efforts led to significant stabilization, with an increase in the melting point by up to 13.2 °C, which translated into better resistance to organic solvents and enhanced compatibility with material coatings. In contrast, MacQ mutants, although destabilized, showed substantial improvements in kinetic properties—more than a 10-fold increase in activity against specific AHLs like N-butyryl-L-homoserine lactone and N-hexanoyl-L-homoserine lactone. These enhanced variants demonstrated improved quorum-quenching abilities, which were further validated through biosensor models and their effectiveness in inhibiting virulence factor production in *P. aeruginosa*.

### Extracellular DNA (eDNA)-degrading enzymes

#### DNase I

Deoxyribonuclease (DNase) is an endonuclease enzyme that targets phosphodiester bonds near pyrimidine bases, resulting in polynucleotides with 5’-phosphate termini. This enzyme is commonly employed to eliminate DNA contamination from protein and nucleic acid preparations [75]. Additionally, DNase has been shown to enhance the antibiotic susceptibility of pathogens embedded within biofilms during infections [75]. DNase I targets the extracellular DNA (eDNA) within the biofilm matrix. The presence of eDNA is crucial for biofilm stability, acting as a structural scaffold that binds bacterial cells together. By degrading eDNA, DNase I disrupts this matrix, leading to a reduction in biofilm integrity [76]. The study of Quan et al. [75] mentioned that the combined application of DNase I and lysostaphin exhibited a synergistic effect, significantly enhancing the disruption of biofilms compared to either agent used alone. This synergy is attributed to DNase I breaking down the protective matrix, which facilitates deeper penetration of lysostaphin into the biofilm and allows more effective bacterial cell lysis. DNase I was also shown to effectively disrupt the biofilms of *L. monocytogenes*, and *Campylobacter*, indicating that extracellular DNA (eDNA) plays a crucial role in the integrity of these biofilm structures and could be a promising target for biofilm control in these pathogenic organisms [29, 53].

Bacteria such as *Serratia marcescens*, and *S. aureus* have been reported to produce DNase, which can be harnessed for antibiofilm applications [77, 78]. The study of Khwen [78] reported that DNase was successfully extracted, yielding a crude enzyme activity of 38 U/mL and a specific activity of 253.3 U/mg. The purification process involved ammonium sulfate precipitation at 65–85% saturation, followed by ion exchange chromatography using CM cellulose and subsequent gel filtration with Sephadex G-150. Post-purification, the DNase exhibited an enhanced activity of 42 U/mL and a significantly increased specific activity of 4200 U/mg. The enzyme displayed optimal catalytic activity at pH 8 and demonstrated stability across a broad pH range, maintaining 100% activity at pH 8, 90% at pH 9, and 86% at pH 10. The optimal temperature for DNase activity was identified as 37 ºC, with maximum stability also observed at this temperature. Enzyme activity was notably enhanced in the presence of 10 mM concentrations of metal ions, including MnCl₂, KCl, NaCl, MgCl₂, and CaCl₂. The molecular weight of DNase, determined via gel filtration, was approximately 19 kDa.

### Lipid-degrading enzymes

#### Lipase

Microbial lipases are unique enzymes that exhibit diverse characteristics such as alkalinity, acidophilicity, thermotolerance, and thermophilicity. The primary advantage of using microbial sources for lipase production is the ability to manipulate these enzymes to possess specific desired traits, making the process both efficient and cost-effective for large-scale manufacturing.

Lipase enzyme can degrade biofilm extracellular polymeric substances (EPS), a key structural component of biofilms. Prabhawathi and co-workers [39, 79, 80] evaluated the antibiofilm efficacy of the lipase-immobilized matrix against common biofilm-forming pathogens such as *S. aureus* and *P. aeruginosa*. They reported that a significant reduction in biofilm formation was recorded which was attributed to the enzymatic degradation of the EPS matrix by lipase which hydrolyzes the lipid components within the EPS. This disruption of the biofilm’s structural integrity enhances the susceptibility of bacteria to antimicrobial agents and hinders their ability to form resistant biofilm communities. Therefore, the study highlighted the potential use of lipase as a coating material for catheters, implants, and other indwelling medical devices.

The utilization of readily available, low-cost lipase substrates derived from microbial sources enhances the economic feasibility of enzyme applications. However, to maximize enzyme yield, it is essential to establish optimal media compositions and culture conditions. Lipase biosynthesis and microbial growth typically require carbon sources like sugars, alongside organic or inorganic nitrogen sources [80]. For optimum production of lipase, a chemical mutagenesis approach could be followed using ethidium bromide as an inducer. Enhancing lipase yield on a larger scale necessitates the optimization of multiple parameters, including the manipulation of the culture medium. Several physicochemical factors such as pH, temperature, incubation duration, and the availability of carbon and nitrogen sources significantly influence enzyme production. Submerged fermentation has been extensively studied for lipase biosynthesis, focusing on optimizing the associated physicochemical conditions [79].

In microbial biotechnology, statistical design methods, particularly Central Composite Designs (CCDs), are widely employed to enhance process optimization. The Response Surface Methodology (RSM) is utilized to identify and optimize responses influenced by various factors through systematically designed experiments. This method evaluates interactions between variables in a quadratic framework, predicting responses and assessing model validity. For industrial applications, RSM simplifies the optimization process by predicting and analyzing the combined effects of different variables without necessitating extensive experimental trials. Response surface contour plots are commonly used to visually represent parameter interactions, thereby facilitating the maximization of microbial enzyme production.

### Other enzymes

#### Cellobiose dehydrogenase (CDH)

Cellobiose dehydrogenase (CDH) is an antimicrobial extracellular enzyme produced by various wood-degrading fungi. Using a diverse range of electron acceptors, it converts soluble cellodextrins, mannodextrins, and lactose to their corresponding lactones, and produces hydrogen peroxide using oxygen as an electron acceptor [24, 40]. Based on the stability of CDH to produce H_2_O_2_ in the presence of cellobiose (disaccharide), a unique in situ antibiofilm and antibacterial system has effectively developed [24].

*Aspergillus niger*-isolated cellobiose dehydrogenase enzyme (CDH) was used as an antibiofilm agent on clinical isolates of *S. epidermidis* and *P. aeruginosa*. This enzyme destabilizes the biofilm and increases the amount of terminal-reducing sugars by hydrolyzing polysaccharides [24].

In 1999, a study was published detailing the first instance of CDH enzyme synthesis in an *A. niger* species. Using the zymogram technique, the researcher demonstrated that the CDH enzyme in the *A. niger* fungus is an extracellular enzyme that is secreted into the culture medium [24]. Also, *Sclerotium rolfsii* is recognized as a reliable source of CDH. This fungus was maintained on glucose-maltose Sabouraud agar plates that were cultured at 30 °C for 5–7 days after being inoculated with a piece of overgrown agar (diameter: 1 cm). For the production of CDH, a medium containing 43 g/L α-Cellulose,80 g/L peptone from meat 2.5 g/L NH4NO3, 1.5 g/L MgSO4 × 7H2O, 1.2 g/L KH2PO4, 0.6 g/L KCl, and 0.3 ml/L trace element solution (1.0 g/L ZnSO4 x H2O, 0.3 g/L MnCl2 × 4H2O, 3.0 g/L H3BO3, 2.0 g/L CoCl2 × 6H2O, 0.1 g/L CuSO4 × 5H2O, 0.2 g/L NiCl2 × 6H2O and 4.0 ml/L conc. H2SO4). Was inoculated with several agar plugs (approximately 1 cm^2^ diameter) taken from 4-day-old agar cultures. For optimum production of CDH; a relatively complex growth media may be reduced to a simple one that only contains cellulose, meat-derived peptone, and trace elements [38]. Enzyme production is adversely affected by NH_4_NO_3_ and KH_2_PO_4_ salts. On the other hand, KCl and MgSO_4_ do not appear to have any effect on CDH production as there was no discernible difference in the production of the enzyme when these salts were not present [38]. A pH-stat approach was used, which involved measuring the pH every day and readjusting it to 5.5 with 5 M NaOH [38].

DCPIP screening medium was made ready a solid screening medium. Cellobiose, the Cezapek dox agar, chloramphenicol, and cellulose were all included in this medium, along with 2, 6-dichloroindophenol. The wells that were prepared in each plate were inoculated with 20 µl of spore suspension of fungal strains. Plates were stored at 25 °C for seven to ten days. After *A. niger* produced glutathione sulfate dehydrogenase and reduced the amount of 2, 6-DCPIP in the culture, a visible halo was created around the colony that was closely correlated with the amount of enzyme produced [24].

#### Efficacy & benefits of enzymatic techniques in antibacterial biofilm disruption

Enzymatic techniques have shown promising potential for disrupting bacterial biofilms and improving the treatment of biofilm-associated infections, which are often resistant to conventional antimicrobial therapies. The use of enzymes such as glycosidases, proteases, and deoxyribonucleases (DNases) is being explored to degrade the extracellular matrix (ECM) of bacterial biofilms, allowing better penetration of antibiotics and promoting biofilm dispersal. Below is a detailed discussion of the benefits, drawbacks, and efficacy of enzymatic techniques for disrupting bacterial biofilms [81]. First, the use of this techniques result in the enhancement of antibiotic penetration and efficacy mainly by biofilm matrix degradation as the extracellular matrix of bacterial biofilms is primarily composed of polysaccharides, proteins, and extracellular DNA (eDNA). Enzymes such as glycosidases (e.g., β-glucanases, mannanases) break down polysaccharides, proteases degrade matrix proteins, and DNases break down eDNA. Disrupting these matrix components enhances the penetration of antibiotics, allowing the drugs to reach bacterial cells more effectively [81]. Enzymes can prevent the initial attachment of bacteria to surfaces, which is a critical step in biofilm formation. For example, mannanases can disrupt the adhesion of *P. aeruginosa* and *S. aureus*, which are both common biofilm-forming pathogens by degradation of polysaccharides that facilitate bacterial adhesion, thus preventing biofilm formation [82]. Also, Enzymes can interfere with bacterial communication, reducing the virulence and biofilm-forming capacity of pathogens like *P. aeruginosa* [83].

### Drawbacks of of enzymatic techniques

Although enzymatic techniques for antibiofilm treatment show promise in dispersing biofilms, it presents several drawbacks that can limit their effectiveness. First; variation in enzyme specificity and efficacy as enzymes like DspB and lysostaphin demonstrate varying effectiveness against different biofilm compositions, which can lead to inconsistent results [84]. Some enzymes may not effectively degrade all components of the biofilm matrix, resulting in incomplete biofilm removal [85]. Second disadvantage can be described in terms of lack of synergy as many enzyme combinations fail to exhibit synergistic effects, limiting their overall efficacy [85]. In addition, enzymatic activity can be influenced by environmental factors such as pH and temperature, which may not always be optimal for enzyme function [86].

Despite these drawbacks, enzymatic techniques remain a valuable area of research, particularly when combined with other antimicrobial strategies. However, further studies are needed to optimize enzyme formulations and application methods for more effective biofilm control.

### Challenges or limitations of enzyme-based treatments

The stability, affordability, and efficacy of the enzymes used are the main challenges facing enzyme-based therapies for biofilm infections, despite the fact that enzymes may degrade the extracellular polymeric substances (EPS) that protect biofilms. The efficacy of natural enzymes in biofilm destruction may be diminished due to their poor stability in physiological settings [87]. More robust enzyme formulations must be developed since environmental variables like pH and temperature might further impair enzyme activity [68]. Taking into consideration the high cost of producing and purifying enzymes can limit their widespread application in clinical settings [87]. Because of its thick EPS matrix, mature biofilms are frequently more durable and may be difficult for enzymes to break through [69]. Although some research indicates that biofilm biomass can be reduced, total eradication is still difficult, especially for complex biofilm formations [88]. Despite these challenges, ongoing research into enzyme functionalization and nanoparticle delivery systems shows potential for enhancing the efficacy of enzyme-based treatments against biofilms [89]. However, the need for further optimization and cost reduction remains critical for practical applications.

### Potential bacterial resistance to enzymatic techniques

The emergence of bacterial resistance to antibiofilm microbial enzymes is a growing concern, particularly as these enzymes have become promising tools in biofilm disruption. Bacteria can develop resistance mechanisms similar to those seen with traditional antibiotics. For example, biofilms exhibit complex defense strategies like altering the expression of efflux pumps, modifying quorum sensing pathways, and enhancing extracellular polymeric substance (EPS) production. These adaptations can shield bacterial cells from enzymatic degradation by increasing biofilm density or reducing enzyme penetration. Additionally, some bacteria may acquire mutations that alter biofilm matrix components, making them less susceptible to enzymatic action. Persistence cells, which are dormant subpopulations within biofilms, can also survive enzymatic treatment and lead to biofilm reformation after treatment cessation. These factors highlight the need for multi-targeted approaches when employing antibiofilm enzymes as a treatment strategy [90].

### Recent application of microbial enzymes

Recent research demonstrates how certain enzymes may break down biofilms, improve the effectiveness of antibiotics, and offer environmentally acceptable alternatives. Proteases and α-amylase are two examples of enzymes that are efficient in breaking up biofilms on marine surfaces. By attacking the biofilm matrix, these enzymes cause biopolymers to break down and bacterial signaling to be disrupted, which can greatly lessen biofouling on ship hulls and aquaculture equipment [91]. A research on *P. aeruginosa* biofilms in burn wounds showed that tobramycin was more effective when the quorum-quenching enzyme AidHA147G and the hydrolase PslG were combined. This combination demonstrated a potential strategy for treating persistent infections by reducing tissue damage and inflammation in addition to inhibiting the production of biofilms [92]. Also, recent studies reveal that the immobilised enzymes, such pectinase and amylase, have better antibiofilm qualities than their free counterparts. Their stability and reusability are improved by this immobilisation, which makes them effective biofilm removal agents for a range of industrial applications [92]. A study on *Vibrio parahaemolyticus* revealed that a combination of lipase, cellulase, and proteinase K significantly inhibited biofilm formation, achieving an 89.7% inhibition rate. This approach not only disrupted the biofilm matrix but also suppressed the expression of related genes, indicating its potential in food safety applications [93].

While enzymatic techniques show great promise, challenges remain in optimizing enzyme combinations and understanding their interactions in complex environments. Further research is essential to fully harness their potential in diverse applications.

### Future advancements in enzyme engineering or synthetic biology

Future advancements in enzyme engineering and synthetic biology hold tremendous potential for developing novel antibiofilm strategies. Traditional antimicrobial agents often struggle against biofilms due to their complex extracellular matrix, which provides a protective barrier for the embedded bacteria. Engineered enzymes, particularly those targeting the biofilm matrix components like polysaccharides, extracellular DNA (eDNA), and proteins, have shown promise in disassembling biofilms [90].

Enzymes that degrade biofilm components are promising candidates for biofilm control. Using synthetic biology, researchers have developed genetically modified bacteria capable of producing enzymes with enhanced activity against biofilms. Additionally, enzymes like DNase I, dispersin B, and proteases can be engineered to target specific biofilm matrix components, facilitating biofilm disintegration. The use of CRISPR-based systems to target and degrade specific biofilm-forming genes in situ is another promising approach [35].

Kim et al. [94] developed a new class of chimeric lytic enzymes using a modular assembly technique, which involves engineering enzyme constructs with enhanced antimicrobial properties by combining various functional domains with enhanced activity and specificity against MRSA biofilm.

Quorum sensing (QS) is a cell-density-dependent communication mechanism that regulates biofilm formation and virulence in many bacterial species. Synthetic biology strategies target QS systems to interfere with biofilm development. One approach involves engineering bacteria or designing synthetic circuits that produce quorum quenching enzymes like lactonases and acylases. These enzymes degrade signaling molecules (e.g., acyl-homoserine lactones), thereby, disrupting communication and preventing biofilm formation [95].

### Concluding remarks and future prospectives

Enzymes provide a sustainable alternative to traditional chemical sanitizers, minimizing environmental impact while effectively managing biofilms. Future studies on enzymes as antibiofilm agents show promise, with an emphasis on how they can prevent the production of biofilms and improve the effectiveness of treatments for illnesses linked to biofilms. The potential of enzymes, especially glycoside hydrolases and carbohydrases, to target the exopolysaccharide matrix of biofilms is being investigated as a more environmentally friendly option to conventional techniques.

More research is being done on the use of antimicrobial enzymes that target various bacterial cellular components and biofilm development to control germs in the production of food, medical care, and environmental protection. It is evident that a combination of enzymes (proteases, polysaccharide-degrading enzymes, DNases, and anti-quorum sensing enzymes) can act in a complementary manner to prevent the formation of biofilms or to destroy existing microbial biofilms, even though some enzymes are effective as stand-alone antimicrobial agents.

Utilizing all the biotechnology advancements including protein engineering, synthetic biology, domain swapping and gene shuffling, bioinformatics, metagenomics, and large-scale DNA sequencing technologies, to create new, more effective antimicrobial and anti-biofilm enzymes is the challenge for this field’s future research endeavors.

## Data Availability

No datasets were generated or analysed during the current study.
